# Comparing the Effects of Exoskeletal-Type Robot-Assisted Gait Training on Patients with Ataxic or Hemiplegic Stroke

**DOI:** 10.3390/brainsci12091261

**Published:** 2022-09-17

**Authors:** Sungsik Son, Kil-Byung Lim, Jiyong Kim, Changhun Lee, Sung II Cho, Jeehyun Yoo

**Affiliations:** 1 Department of Rehabilitation Medicine, Inje University Ilsan Paik Hospital, Juhwa-ro 170, Ilsanseo-gu, Goyang-si 10380, Gyeonggi-do, Korea; 2 Department of Rehabilitation Center, Inje University Ilsan Paik Hospital, Juhwa-ro 170, Ilsanseo-gu, Goyang-si 10380, Gyeonggi-do, Korea

**Keywords:** robotics, robot-assisted gait training, stroke, ataxia, hemiplegia, robotic rehabilitation, EXOWALK^®^

## Abstract

This study aimed to discover the effects of robotic rehabilitation utilizing an exoskeletal-type robot-assisted gait training (RAGT) device on patients with ataxic and hemiplegic stroke and to compare its effectiveness between the two groups. This was a retrospective study, and the electronic charts of 22 patients who underwent RAGT treatment from October 2019 to June 2021 were reviewed. Patients were divided into ataxic and hemiplegic groups based on their symptoms. The clinical outcome measures included the Berg balance scale (BBS), functional ambulation category (FAC), and mobility subcategories of the modified Barthel Index (MBI-m). Outcome measures were reviewed at two points within 48 h, before and after RAGT with EXOWALK^®^, a type of exoskeletal robot. After the RAGT sessions, total patients in both ataxic and hemiplegic groups demonstrated statistically significant improvements in BBS (*p* < 0.0001, *p* = 0.002, and *p* = 0.005, respectively) and MBI-m (*p* < 0.0001, *p* = 0.002, and *p* = 0.011, respectively). Additionally, FAC after RAGT was significantly improved (*p* = 0.0056). The regression coefficient of the number of RAGT treatments for BBS changes in the nine subjects was estimated to be 2.45; 3.50 in the ataxic group and 2.26 in the hemiplegic group. The regression coefficient of the number of RAGT treatments for MBI-m changes in the nine subjects was estimated to be 0.16; 4.00 in the ataxic group and −0.52 in the hemiplegic group. Our results suggest that RAGT using an exoskeletal-type robot, EXOWALK^®^, could be effective for improving walking capacity, balance, and daily activities of life in patients with ataxic and hemiplegic stroke.

## 1. Introduction

Stroke, along with malignant neoplasms and heart disease, is one of Korea’s three leading causes of death and can cause severe disability. However, professional and comprehensive rehabilitation starting from the acute stage of stroke can improve functional recovery and minimize disability [1]. Stroke patients experience several symptoms, including paralysis, which can cause gait disturbances. However, ataxia can impair gait ability and daily activities in patients whose paralysis symptoms are not prominent [2].

Ataxia is a neurological feature characterized by a lack of voluntary coordination of muscle movements, leading to gait abnormalities, dysarthria, and ocular palsy. It is a clinical manifestation that indicates dysfunction of the parts of the nervous system that coordinate movements, such as the cerebellum and brainstem [3,4]. Cerebellar strokes are relatively uncommon, accounting for fewer than 10% of all strokes [5], while the brainstem accounts for 10–15% of all strokes [6]. Both cerebellar and brainstem strokes can cause ataxia, and a lack of voluntary coordination of muscle movements can cause gait abnormalities. In patients with ataxia, gait is marked by a shortened stride length, high step pattern, and decreased push-off and veering, which have been closely linked to the severity of the individual’s balance deficits; leading to taxing and unsafe mobility and increased fall risk [7]. A balance deficit combined with dystonia, decreased joint mobility, and loss of proprioception is associated with increased difficulty performing daily activities [8]. Therefore, a comprehensive therapeutic intervention is the primary treatment option [9].

For rehabilitative gait training for patients with stroke, along with conventional therapy, robot-assisted gait training (RAGT) is emerging as effective. It is superior to overground gait training because it provides a highly repetitive intensive training of complex, normal gait cycles to patients while requiring less effort from physical therapists [10]. The robot used for gait training is differentiated into end-effector and exoskeletal-type devices. Exoskeletal devices are equipped with programmable drives or passive elements to move the knee and hip joints during gait phases [10]. Through this action, an exoskeletal-type robot could lead to gait re-education [11]. Many studies have demonstrated the effectiveness of an exoskeletal type of RAGT in patients with stroke [10,12,13]. Nam et al. reported that patients who received exoskeletal RAGT combined with conventional physiotherapy after an ischemic or hemorrhagic hemiplegic stroke had a more significant improvement in their functional ambulatory category than patients who received only conventional gait training [14].

RAGT is known to improve balance and walking ability through high-intensity repetitive training [13]. Therefore, it can be assumed that RAGT may be effective in ataxic patients whose main problem is a balance deficit. However, previous studies on the effects of RAGT have been conducted more on hemiplegic stroke patients than ataxic stroke patients. A previous study evaluated the effect of RAGT using an exoskeletal-type robot for ataxic stroke, but the participants were chronic patients [15]. However, a recent meta-analysis has verified that RAGT could be more effective for balance recovery in acute/subacute stroke patients than in chronic stroke patients [16]. Thus, we think it is necessary to reveal the effectiveness of RAGT on acute/subacute ataxic stroke patients.

EXOWALK^®^ (HMH, Incheon, Korea) is an exoskeletal-type robot. It attaches the patient’s foot, calf, and thigh to the robot and controls joint motions. The two main differences between EXOWALK^®^ and existing treadmill-based exoskeletal-type robots, such as Lokomat^®^, are: (1) EXOWALK^®^ can move on the ground during patient gait training, and this makes the patient feel as if they are walking on the floor, and (2) EXOWALK^®^ does not have a harness for trunk control and does not provide body weight support. Therefore, patients must try to control their trunks during EXOWALK^®^ training. Destabilization training could occur due to fewer constraints and more freedom of movement in the trunk and pelvic joints. Therefore, we may assume that RAGT using EXOWALK^®^ could provide ‘task-specific’ balance training, a concept from Gandolfi et al., to ataxic stroke patients [17].

Considering the lack of RAGT studies on acute/subacute ataxia patients and that EXOWALK could provide task-specific balance training, we proceeded with this analysis. This study aimed to evaluate the effects of RAGT with an exoskeletal-type robot, EXOWALK^®^, in patients with ataxic or hemiplegic stroke, especially for balance, ambulation capacity, and mobility. Through this investigation, we attempted to confirm that RAGT is as effective on ataxic patients as on hemiplegic patients.

## 2. Methods

### 2.1. Patients

**Figure 1:**
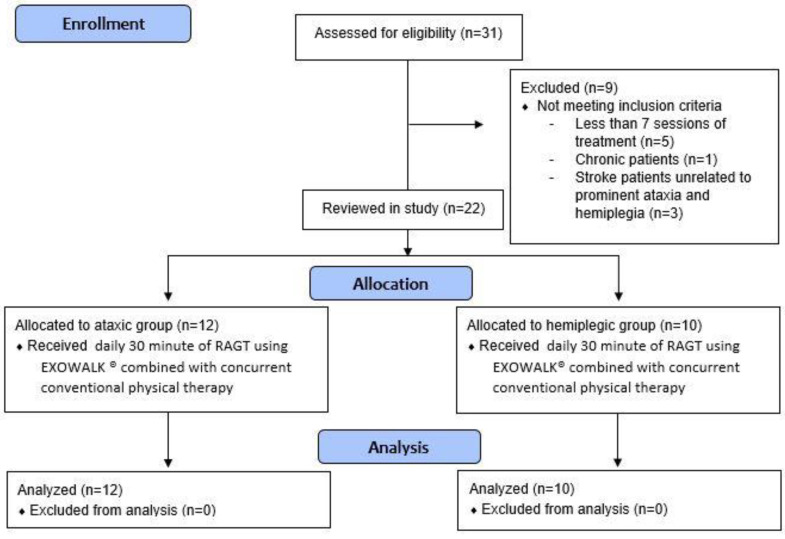
Flowchart of this study.

We reviewed the electronic charts of inpatients in our rehabilitation department between October 2019 and June 2021. The inclusion criteria involved patients: (1) who underwent RAGT with EXOWALK^®^; (2) with acute, subacute ischemic, or hemorrhagic stroke confirmed by brain imaging; (3) with first onset stroke; (4) who were able to walk independently before onset; (5) with FAC 3 or lower; (6) with sufficient cognitive function to obey one-step verbal commands; and (7) aged ≥ 19 years. Exclusion criteria involved patients: (1) with truncal ataxia with motor strength of the extremities less than grade 4, and (2) with hemiplegic stroke with prominent truncal ataxia. Subsequently, we divided the patients into ataxic and hemiplegic groups. Patients with truncal ataxia while controlling their trunk during standing or ambulation or with cerebellar and brainstem lesions confirmed by brain imaging were defined as the ataxic group. Patients with hemiplegic motor deficits were assigned to the hemiplegic group. Thirty-one patients were assessed for eligibility, and nine were excluded because they did not meet the inclusion criteria. Therefore, 12 patients with ataxic stroke and 10 patients with hemiplegic stroke were reviewed in this study (Figure 1). Our Institutional Review Board approved this study (approval number: ISPAIK 2022-01-013).

### 2.2. RAGT with EXOWALK^®^

**Figure 2:**
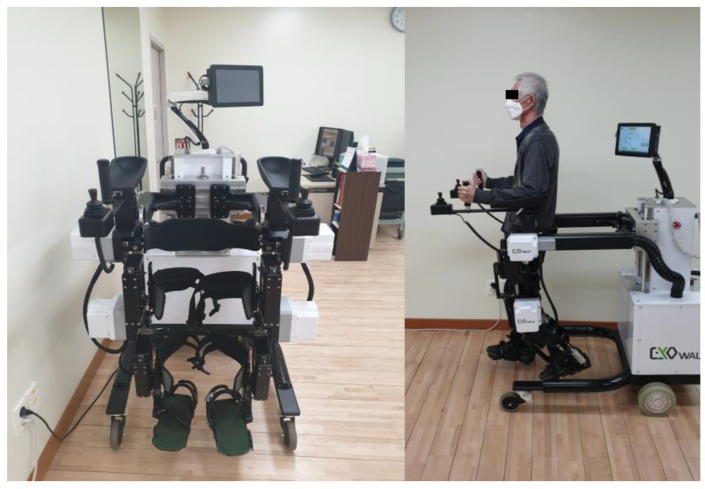
The EXOWALK^®^ (HMH, Incheon, Korea) exoskeletal-type robot.

In this study, we reviewed the electronic charts of patients who underwent RAGT using EXOWALK^®^ (Figure 2). EXOWALK^®^ can move forward, backward, and turn around under the therapist’s control. Cadence (steps/min), gait speed (km/hr), and step length (cm) are adjusted as the patient’s gait ability at each training session. After the training session, the total gait distance (m) and the number of steps are presented.

Patients in both groups underwent daily 30 min of RAGT using EXOWALK^®^ from a minimum of 7 times to a maximum of 16 times during their hospitalization, combined with one hour of concurrent conventional physical therapy. Conventional physical therapy consists of joint range of motion, muscle strengthening, and functional exercises, including sit-to-stand, sitting, and standing balance exercises.

### 2.3. Outcome Measures

The electronic chart review measured the outcomes of the BBS, FAC, and mobility subcategories of the modified Barthel Index (MBI-m) within 48 h before and after RAGT. The BBS is a useful scale to evaluate the falling risk, focusing on static and dynamic balance, and includes 14 tasks with a maximum score of 56 [18]. The FAC is a 6-point functional walking category. It evaluates ambulation ability, determines how much human support the patient requires when walking, and has good reliability and concurrent and predictive validity [19]. The MBI is a valid and reliable index used to measure a patient’s daily activities. Ten items were related to the degree of independence from help: feeding, dressing, personal hygiene, bathing, toilet transfer, bladder control, bowel control, chair or bed transfers, ambulation, and stair climbing [20]. We used only the MBI-m, which includes ambulation, transfer, and stair climbing. Outcome measures at two points were reviewed: within 48 h before and after RAGT.

### 2.4. Statistical Analysis

Demographic information of subjects included in the study was collected for the total subjects and each group. The average and standard deviation were presented as continuous variables; frequency and ratio were calculated as categorical variables.

For continuous variables, after determining whether the data followed a normal distribution using the Shapiro–Wilk test, we used non-parametric tests to analyze the data. The Wilcoxon signed-rank test was used to compare the BBS and MBI-m pre- and post-RAGT, and the Bowker’s symmetry test was used to compare the FAC pre- and post-RAGT.

To confirm the effectiveness of RAGT treatment, a short-term estimation was performed for BBS and MBI-m, considering the number of RAGT sessions and the onset days.

The ordinary least squares method linear regression model was used to estimate the effect of the number of RAGT sessions on BBS and MBI-m variations. Considering the limitations on the number of subjects included in this study and short-term estimation, the level of statistical significance was not considered. The regression model was set as the number of RAGT sessions and disease groups for the independent value and the changes in BBS and MBI-m for the dependent value. There were 22 subjects included in this study. However, we selected subjects to be included in the regression analysis. In general, it is known that patients in the acute phase, within 30 days of onset, are highly likely to recover their function. Therefore, subjects treated within 30 days were selected for accurate regression analysis. A regression analysis was conducted on 9 patients (5 patients ataxic, 4 patients hemiplegic), excluding individuals who were not treated continuously due to pneumonia, urinary tract infection, and dizziness within the acute period. In addition, the regression coefficient for the number of RAGT sessions for each group was estimated by considering the difference in paralysis characteristics between ataxia and hemiplegia groups.

SPSS version 25.0 (IBM Corp., Armonk, NY, USA) was used to perform statistical analyses. The statistical significance was a two-sided test with a significance level of less than 5%.

## 3. Results

### 3.1. Patients’ Characteristics

Table 1 presents the patients’ baseline characteristics. Patient age, time from onset, sex, etiology, and RAGT treatment duration did not differ significantly between the groups.

### 3.2. Outcome Measures

**Figure 3:**
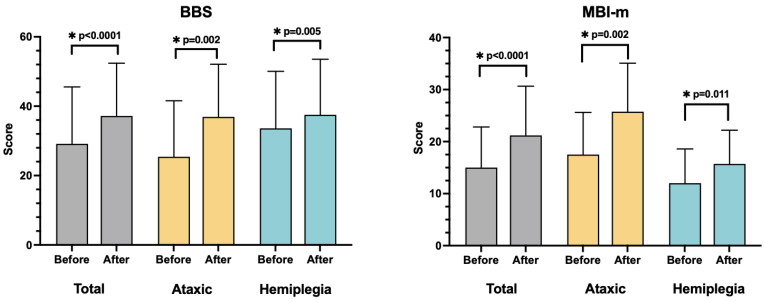
Comparative analysis of the BBS and MBI-m within the groups. All outcome measures improved significantly after RAGT in total subjects and both groups. * *p* < 0.05, statistically significant.

After RAGT, the ataxic and hemiplegic groups demonstrated significant improvements in the BBS, FAC, and MBI-m (Table 2 and Table 3 and Figure 3). One patient with initial FAC 0, and another with FAC 1, did not improve after RAGT. However, in other patients, FAC significantly improved after RAGT (Table 3).

The regression coefficient of the number of RAGT treatments for BBS changes in the nine subjects was estimated to be 2.45; 3.50 in the ataxic group and 2.26 in the hemiplegic group. The regression coefficient of the number of RAGT treatments for MBI-m changes in the nine subjects was estimated to be 0.16; 4.00 in the ataxic group and −0.52 in the hemiplegic group (Table 4). The regression coefficient of the hemiplegic group was a negative value, but it is close to 0 and assumed to be due to the small sample size.

## 4. Discussion

Generally, the primary purpose of gait rehabilitation after stroke is to increase the patient’s independence by improving the functional level, thereby improving their quality of life. However, even if paralysis symptoms are not evident after stroke, the presence of ataxia affecting balance ability, postural stability, and coordination of limbs can interfere with the patient’s gait and daily activities [7].

Few studies have discussed the effects of rehabilitation treatment in patients with ataxic stroke. Januário et al. revealed that a training program using force platform visual biofeedback improved objective measures of bilateral postural stability in 38 patients with hemiplegia and/or ataxia after stroke [21]. Bultmann et al. randomized 23 cerebellar infarction patients; half underwent treadmill training 2 weeks after enrollment. After 2 weeks of treadmill training, the international cooperative ataxia rating scale (ICARS) had no significant difference in scores or subscores between the groups [22].

To the best of our knowledge, this is the first study to report the effects of RAGT using EXOWALK^®^ for patients with ataxia. Balance ability is crucial for regaining ambulation and is the main obstacle for patients with ataxic stroke in managing their independence in daily living [23]. In this study, we used BBS to evaluate the patients’ balance abilities, FAC to evaluate ambulation ability, and MBI-m to evaluate an individual’s ability to perform daily activities. The results demonstrated that exoskeletal RAGT improved FAC, BBS, and MBI-m in ataxic and hemiplegic groups.

Patients with ataxic stroke had sufficient muscle strength in their extremities compared to those with hemiplegic stroke. However, owing to impaired voluntary coordination of muscle movements, achieving independence in gait and daily activities is difficult. Therefore, for safe gait training, limb coordination assistance is needed. Unlike the end-effector-type robot, which does not have a fixed part of the lower extremities other than the footrest, the exoskeletal-type robot has an axis-aligned fixed anatomical axis that anchors the patient’s knee and hip joints. The limbs are fixed and moved; hence, it could offer an effective and safe external force for patients with ataxic stroke to practice gait with higher intensity.

This is supported by studies that reported the effectiveness of exoskeletal-type RAGT in patients with ataxic stroke. Randomized controlled trials have demonstrated that exoskeletal-type RAGT improves the functional level and gait-related values more than conventional gait training [14,24]. Few studies have reported RAGT in patients with ataxic stroke. However, Jung et al. conducted an end-effector-type RAGT in 19 patients with ataxic stroke [25]. They suggested that RAGT could contribute to further improvement in walking ability and balance in patients with ataxic stroke. Additionally, dos Santos et al. discovered that RAGT using an exoskeletal-type device, Lokomat^®^, improved balance and functional independence in patients with chronic ataxic stroke [14].

RAGT has advantages over conventional physiotherapy because it offers higher intensity and numerous repetitions to achieve functional motor relearning [26]. EXOWALK^®^ allows patients to experience accurate normal gait patterns and enables early gait training with advanced intensity compared to gait training supported by a physiotherapist. For example, in a 30 min training session, the patient could walk with the EXOWALK^®^ for more than 600 steps with a maximal velocity of 2.3 km/hr. Unlike Lokomat^®^, an exoskeletal-type gait robot that supports weight with a harness and trains walking based on a treadmill, EXOWALK^®^ does not support weight with a harness, so patients need to pay more attention to maintaining an upright position. This provides the feeling of walking on the ground.

Moreover, EXOWALK^®^ does not have a pelvic strap; therefore, it assists patients in swaying their pelvises naturally and according to the normal movements of the lower extremities. It also allows for several directions of gait training, such as going forward, backward, and turning left or right. During walk training, the patient feels that they are walking on a road because EXOWALK^®^ moves on the floor. This gives patients more motivation and confidence to walk with high satisfaction [14]. The various characteristics of EXOWALK^®^ described above may improve walking and balance ability in patients with ataxic stroke, consistent with previous studies.

There are two types of gait training robots: exoskeletal and end-effector. The RAGT effects could differ because of how a gait cycle is created and joint movement is controlled. Therefore, a randomized control trial is needed to determine which gait training robots are more effective for patients with ataxic and hemiplegic strokes.

We estimated the number of RAGT sessions and the regression coefficient of the outcome change through regression analysis. Although the number of subjects was small, it was estimated that BBS and MBI-m could improve according to the number of RAGT sessions. However, a regression analysis study with larger sample sizes will be needed in the future.

This study has some limitations, including its small sample size. Furthermore, it was a retrospective study, meaning that control groups did not receive RAGT in this study. Therefore, we could not reveal whether RAGT with EXOWALK^®^ is superior to conventional physiotherapy. This was a preliminary investigation before conducting a randomized controlled trial in acute/subacute ataxic stroke patients. Despite this limitation, our study results were meaningful. This is the first study to confirm that RAGT with EXOWALK^®^ is as effective in acute/subacute ataxic patients as in hemiplegic patients. Well-designed randomized controlled trials with larger sample sizes and proper control groups are needed to reveal the effectiveness of RAGT on ataxic stroke.

## 5. Conclusions

RAGT using an exoskeletal-type robot, EXOWALK^®^, could be effective for improving walking capacity, balance, and daily activities in patients with acute, subacute ataxic and hemiplegic stroke. Further well-designed, prospective randomized controlled trials are needed in the future to confirm the effectiveness of exoskeletal-type RAGT in the treatment of acute and subacute ataxic stroke.

## Figures and Tables

**Table 1:** Patients’ baseline characteristics.

	Total (*n* = 22)	Ataxic Group (*n* = 12)	Hemiplegic Group (*n* = 10)	*p*-Value
Age (years)	65.5 (62.0–74.8)	63.5 (61.0–73.0)	69.0 (64.3–74.8)	0.346
Time from onset (days)	21.0 (15.5–53.3)	24.5 (17.0–39.5)	16.0 (12.3–63.8)	0.628
Sex				
Male	16	8	8	
Female	6	4	2	0.646
Etiology				
Ischemic	19	10	9	1.000
Hemorrhagic	3	2	1	
RAGT treatment times	9.5(8.0–11.0)	8.5(8.0–11.0)	10.0(7.5–11.8)	0.539

Values are presented as median (interquartile range) or number. RAGT, robot-assisted gait training.

**Table 2:** Comparative analysis of the BBS and MBI-m within the groups.

	Total (*n* = 22)			Ataxic Group (*n* = 12)			Hemiplegic Group (*n* = 10)		
	Before	After	*p*-Value	Before	After	*p*-Value	Before	After	*p*-Value
BBS	29.14 ± 16.43	37.18 ± 15.21	<0.0001 *	25.42 ± 16.15	36.92 ± 15.19	0.002 *	33.60 ± 16.45	37.50 ± 16.04	0.005 *
MBI-m	15.00 ± 7.81	21.18 ± 9.47	<0.0001 *	17.50 ± 8.12	25.80 ± 9.31	0.002 *	12.00 ± 6.58	15.70 ± 6.48	0.011 *

Values are presented as mean ± SD. BBS, Berg balance scale; MBI-m, mobility subcategories of the modified Barthel Index. * *p* < 0.05, statistically significant.

**Table 3:** Comparative analysis of the FAC within groups.

	Total (*n* = 22)			Ataxic Group (*n* = 12)		Hemiplegic Group (*n* = 10)	
	*n* (%)	95% CI	*p*-Value	*n* (%)	95% CI	*n* (%)	95% CI
Subjects with FAC Improvement	20 (90.9)	(70.8, 98.9)	0.0001 ^#^	12 (100.0)	-	8 (80.0)	(44.4, 97.5)
Pre/Post	**0**	**1**	**2**	**3**	**4**	**Total**	***p*** **-value**
0	1	2	4	0	0	7	0.0056 ^§^
1	0	1	2	2	2	7	
2	0	0	0	4	4	8	
3	0	0	0	0	0	0	

FAC, functional ambulation category category. ^#^ *p* < 0.05, statistically significant in Equivalence Test for Binomial Proportion. ^§^ *p* < 0.05, statistically significant in Bowker’s Symmetry Test.

**Table 4:** Linear regression analysis of the effect of the number of RAGT sessions on BBS and MBI-m.

		Parameter	Estimate	SE	*p*-Value
BBS	Total	Intercept	−15.43	12.02	0.2464
		Count	2.45	1.44	0.1393
		Ataxic vs. Hemiplegic	9.44	2.87	0.0167
	Ataxic	Intercept	−14.00	34.65	0.7132
		Count	3.50	4.55	0.4977
	Hemiplegic	Intercept	−13.89	10.73	0.3249
		Count	2.26	1.29	0.2208
MBI-m	Total	Intercept	3.15	13.16	0.8188
		Count	0.16	1.57	0.9206
		Ataxic vs. Hemiplegic	6.01	3.15	0.1048
	Ataxic	Intercept	−20.00	37.22	0.6284
		Count	4.00	4.89	0.4731
	Hemiplegic	Intercept	8.78	7.60	0.3676
		Count	−0.52	0.91	0.6264

BBS, Berg balance scale; MBI-m, mobility subcategories of the modified Barthel Index.

## Data Availability

The data presented in this study are available upon request from the corresponding author. The data are not publicly available because of ethical and privacy restrictions.

## Abbreviations

RAGT	Robot-assisted gait training
BBS	Berg balance scale
FAC	Functional ambulation category
MBI-m	Mobility subcategories of the modified Barthel Index
ICARS	International cooperative ataxia rating scale.
